# Supplementary figures and images for: Modelling microbiome recovery after antibiotics using a stability landscape framework
Source: ISME J. 2019 Mar 15;13(7):1845–56. doi: 10.1038/s41396-019-0392-1 (PMC6591120; doi:10.1038/s41396-019-0392-1)

## Placebo (n=10)

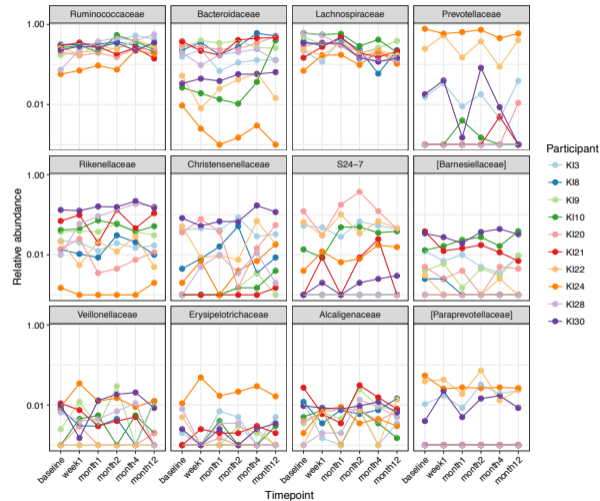

## Clindamycin (n=9)

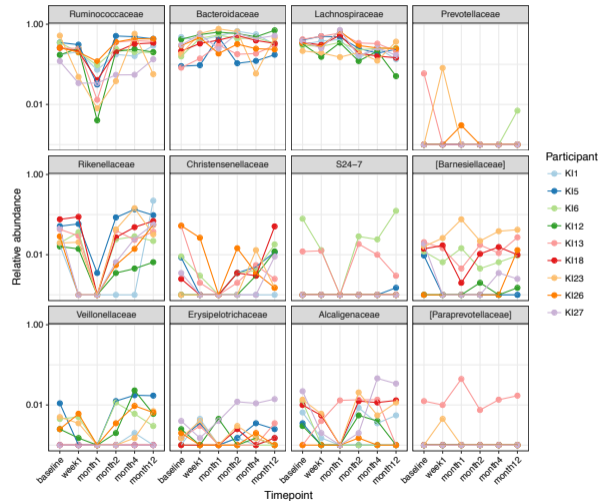

## Ciprofloxacin (n=9)

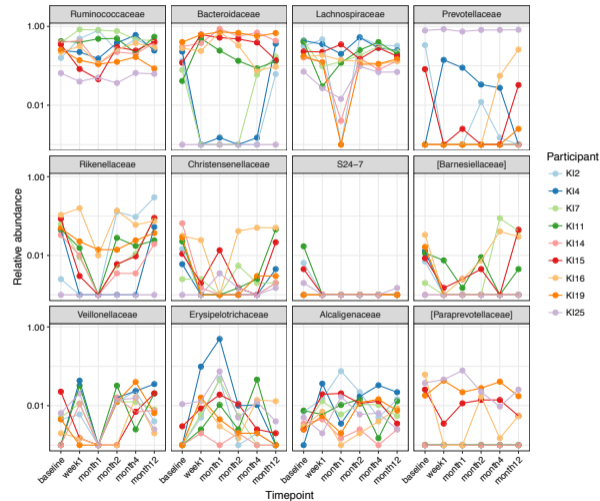

Supplement: Supplementary file 1 — Supplementary Figure 1 [file 41396_2019_392_MOESM1_ESM.pdf]

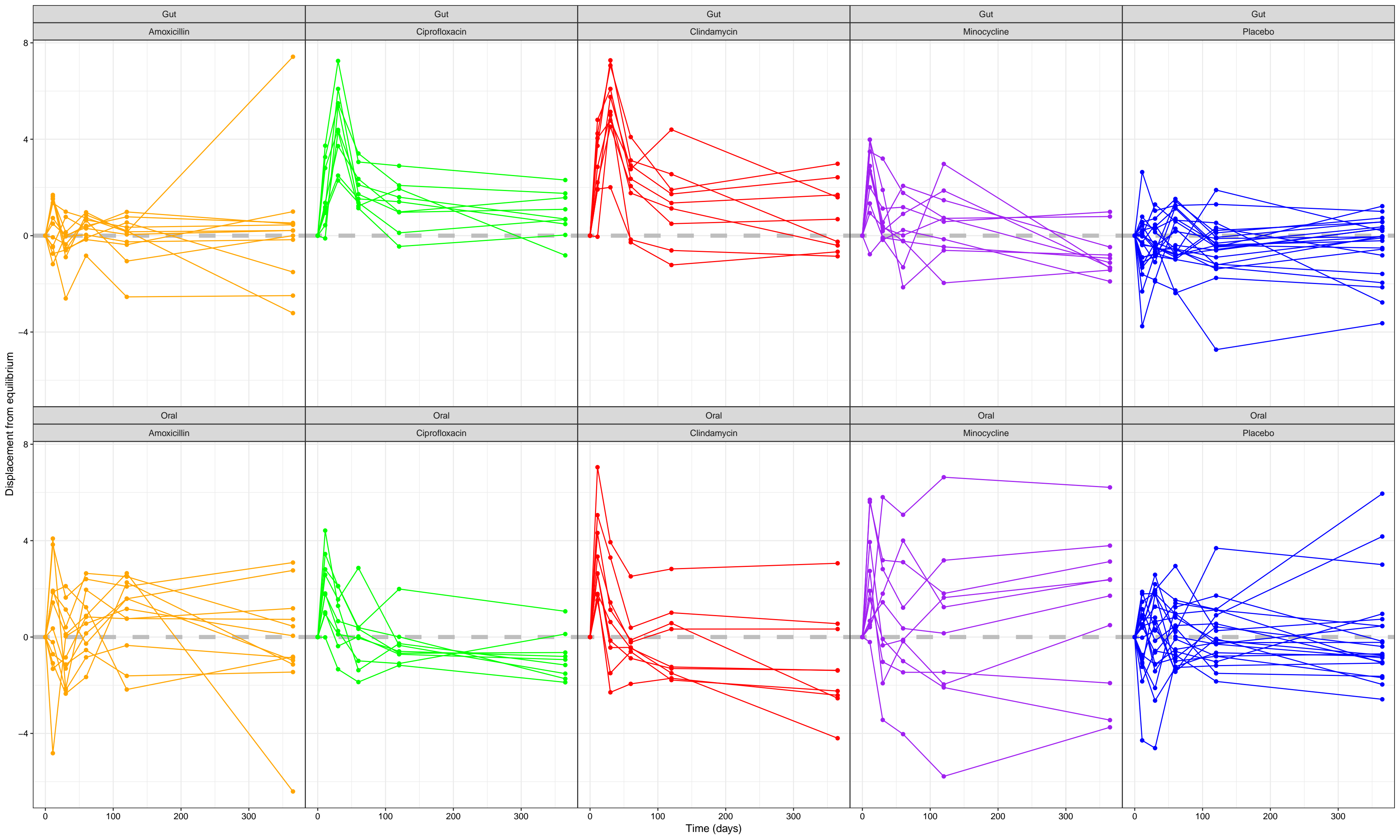

Supplement: Supplementary file 2 — Supplementary Figure 2 [file 41396_2019_392_MOESM2_ESM.pdf]

Model with return to initial equilibrium

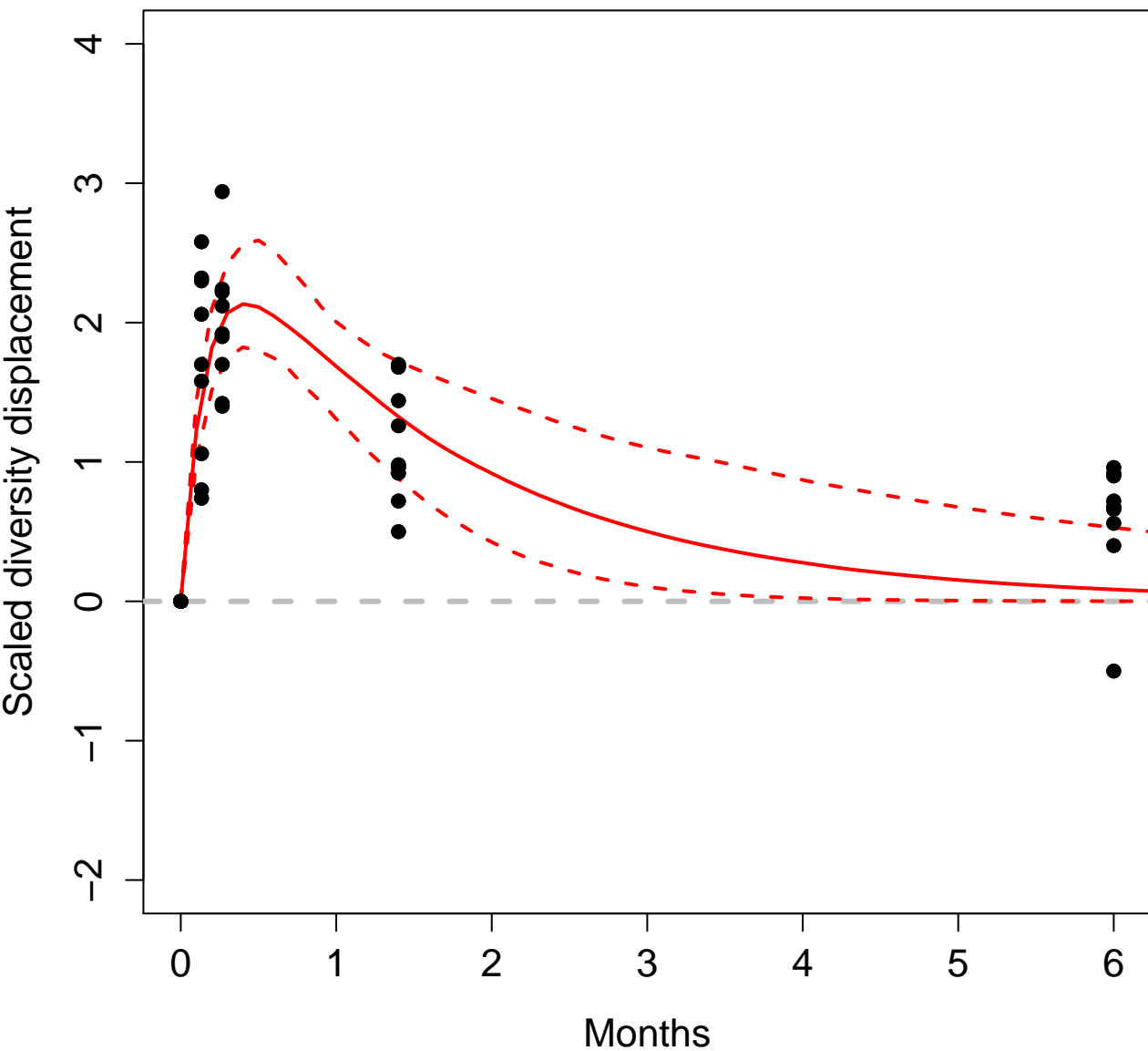

Model with change of equilibrium

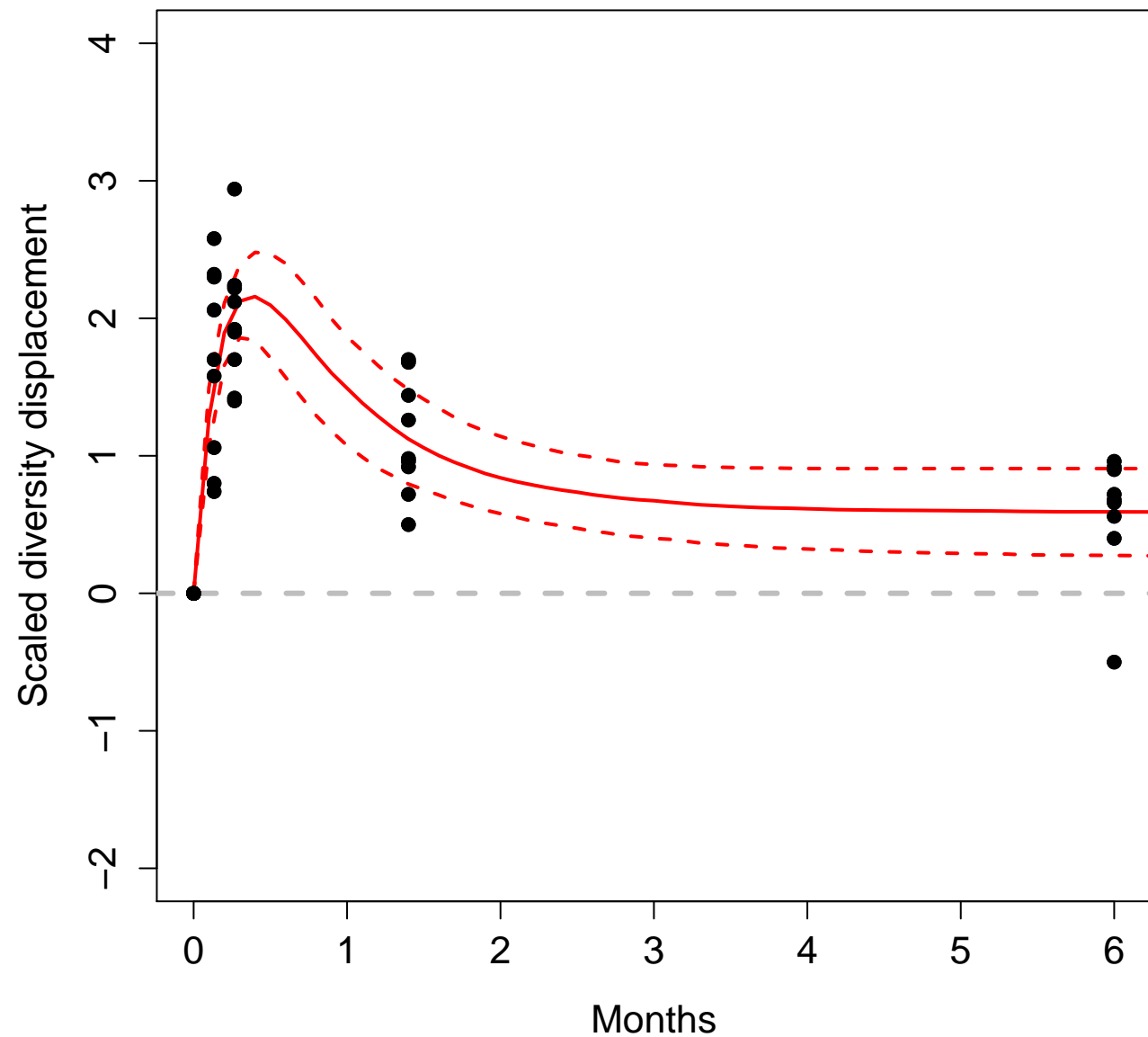

Supplement: Supplementary file 3 — Supplementary Figure 3 [file 41396_2019_392_MOESM3_ESM.pdf]
